# Documentation of corneal epithelial defects with a fluorescein angiographic imaging system

**DOI:** 10.1002/ccr3.2350

**Published:** 2019-07-30

**Authors:** Koushik Tripathy

**Affiliations:** ^1^ ASG Eye Hospital Kolkata India

**Keywords:** corneal epithelial abrasion, corneal ulcer, sodium fluorescein

## Abstract

Fluorescein angiogram mode of fundus camera may be used to document corneal epithelial defects stained with topical fluorescein especially in ophthalmic settings which do not have the facility for slit lamp photography.

This 30‐year‐old woman complained of irritation and pain after removal of contact lens in the left eye. She had Descemet's folds, large corneal epithelial defect with an inferotemporal small epithelial defect, and no infiltrate in the left eye which took staining by topical fluorescein (Figure 1A). The anterior segment image after topical application of fluorescein dye, taken on the fundus camera (Visucam 500, Zeiss) in fluorescein angiogram (FA) mode, showed localized hyperfluorescence at the corneal epithelial defect (Figure 1B).

**Figure 1:**
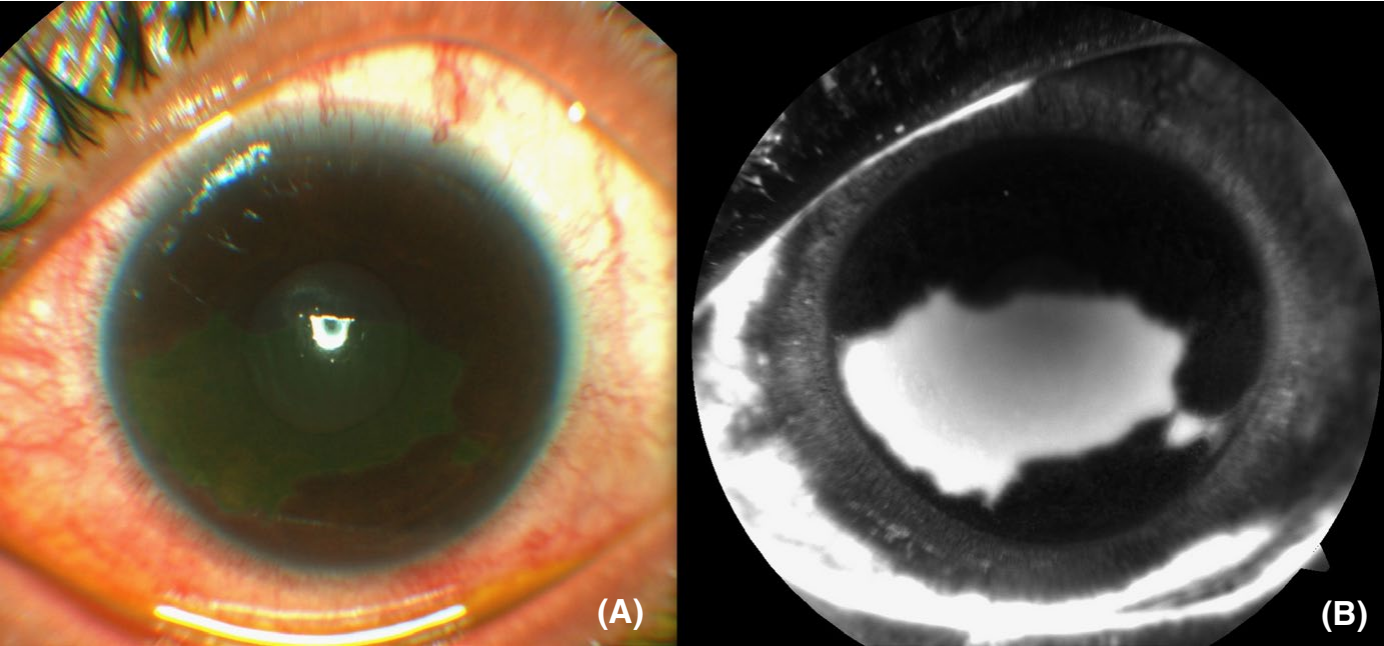
A, Color picture of the anterior segment after staining with topical fluorescein. B, The image of the same eye captured in the fluorescein angiogram mode

Corneal angiography with intravenous administration of sodium fluorescein has been used to evaluate vascular changes in cornea,^1^ iris, conjunctiva, and limbus. Dean and colleagues reported the use of topical fluorescein and FA mode of fundus camera to document corneal disorders such as dendritic corneal ulcer due to herpes simplex virus, punctate staining in dry eye, Fuchs’ endothelial dystrophy with epithelial microcystic edema, and corneal ulcer with some scarring.^2^ The advantages of this imaging include high‐contrast image and lack of “distracting corneal light reflexes.”^2^ Thus, FA mode of fundus camera may be used to document corneal epithelial defects stained with topical fluorescein. This method is very sensitive in our experience and allows us to document even minor and small areas of fluorescein staining.

## CONFLICT OF INTEREST

The author has no conflict of interest with the submission.

## AUTHOR CONTRIBUTIONS

KT: had full access to all data of the manuscript and take responsibility for the integrity of the data. KT: was involved in the acquisition of data, manuscript concept and design, analysis and interpretation of data, drafting of the manuscript, and critical revision of the manuscript for important intellectual content.
